# Unprecedented insights into extents of biological responses to physical forcing in an Arctic sub-mesoscale filament by combining high-resolution measurement approaches

**DOI:** 10.1038/s41598-024-58511-y

**Published:** 2024-04-08

**Authors:** Josefine Friederike Weiß, Wilken-Jon von Appen, Barbara Niehoff, Nicole Hildebrand, Martin Graeve, Stefan Neuhaus, Astrid Bracher, Eva-Maria Nöthig, Katja Metfies

**Affiliations:** 1 Alfred Wegener Institute Helmholtz Centre for Polar and Marine Research (AWI), Am Handelshafen 12, 27570 Bremerhaven, Germany; 2 Alfred Wegener Institute Helmholtz Centre for Polar and Marine Research, Polar Terrestrial Environmental Systems, 14473 Potsdam, Germany; 3 Institute of Environmental Physics (IUP), University Bremen (UB), Otto-Hahn-Allee 1, 28359 Bremen, Germany; 4 Helmholtz Institute for Functional Marine Biodiversity at the University of Oldenburg (HIFMB), Ammerländer Heerstrasse 231, 26129 Oldenburg, Germany

**Keywords:** Biooceanography, Ecosystem ecology, Ocean sciences, Community ecology, Molecular ecology

## Abstract

In Fram Strait, we combined underway-sampling using the remote-controlled Automated Filtration System for Marine Microbes (AUTOFIM) with CTD-sampling for eDNA analyses, and with high-resolution optical measurements in an unprecedented approach to determine variability in plankton composition in response to physical forcing in a sub-mesoscale filament. We determined plankton composition and biomass near the surface with a horizontal resolution of ~ 2 km, and addressed vertical variability at five selected sites. Inside and near the filament, plankton composition was tightly linked to the hydrological dynamics related to the presence of sea ice. The comprehensive data set indicates that sea-ice melt related stratification near the surface inside the sub-mesoscale filament resulted in increased sequence abundances of sea ice-associated diatoms and zooplankton near the surface. In analogy to the physical data set, the underway eDNA data, complemented with highly sampled phytoplankton pigment data suggest a corridor of 7 km along the filament with enhanced photosynthetic biomass and sequence abundances of sea-ice associated plankton. Thus, based on our data we extrapolated an area of 350 km^2^ in Fram Strait with enhanced plankton abundances, possibly leading to enhanced POC export in an area that is around a magnitude larger than the visible streak of sea-ice.

## Introduction

The plankton community structure in the marine realm is highly variable over temporal and geographical scales, related to variability in physicochemical drivers (e.g. temperature, salinity, nutrient availability, sea-ice coverage) and biological processes (phenological responses, advection and migration, population dynamics). Mesoscale hydrographic features (horizontal scale of 10–100 km) have been shown to impact biological and biogeochemical processes in various ways, including the modulation of nutrient supply to the euphotic zone leading to increased primary production^1^, and community turnover due to vertical and horizontal transport^2–4^. Spatial and temporal turnover in species composition and biomass structure can affect food webs via losses and gains of trophic interactions, or by influencing the magnitude of existing interactions^3–6^, leading to high spatio-temporal variability in biogeochemical processes and carbon fluxes. Observations by satellite remote sensing, autonomous profiling floats, and towed instruments, in combination with improved modeling capacities highlighted the ubiquity and ecological relevance of oceanographic features at an even finer scale, the sub-mesoscale (0.1–10 km) scale^1,7–9^. Such sub-mesoscale structures significantly differ in their physical properties from mesoscale structures and in terms of dynamics and impact. They often appear between mesoscale eddies^1,10^ or in the presence of strong horizontal gradients, for instance in the marginal ice zone (MIZ)^11^.

There is growing interest in understanding the role of sub-mesoscale structures in shaping patchiness and variability of ecological networks and biogeochemical processes in order to better understand how ecosystems function, how their functionality varies with space and time, and how they respond to future environmental scenarios. Satellite observations revealed increasing chlorophyll *a* (Chl* a*) concentrations as a function of physical dynamics and intake of nutrients in sub-mesoscale fronts^12,13^. Due to their small spatial and short temporal (hours to weeks) scales, in situ measurements, especially those addressing their impact on biological processes, are, however, difficult. Plankton biodiversity studies still largely depend on point sampling by CTD rosettes or net hauls from research vessels, with distances between stations usually too coarse to resolve the impact of sub-mesoscale features on small-scale plankton variability. Thus, our understanding of the impact of such small-scale dynamics on plankton composition and interactions is currently very limited. Sophisticated automated underway sampling-technology like the Automated Filtration System for Marine Microbes (AUTOFIM) installed on board research vessels^14^ can augment the current constraints with adequate horizontal resolution. Combining such technology with molecular plankton biodiversity studies based on 18S metabarcoding provides high-resolution information on plankton dynamics at various spatio-temporal scales^15^.

Understanding linkages between sub-mesoscale features and ecosystem functionality is particularly important for marine areas, such as Fram Strait, that are frequently impacted by a highly dynamic and turbulent regime of mesoscale features^11,16^. In eastern Fram Strait, warm saline Atlantic water is transported via the West Spitsbergen Current (WSC) northward, while cold and rather fresh Polar water is transported southward in western Fram Strait via the East Greenland current (EGC). A highly dynamic oceanographic regime in the zone between the two currents in combination with the semi-permanent sea-ice edge^11^ and large horizontal density gradients observed in the marginal ice zone (MIZ) in the northern Fram Strait enhance the development of sub-mesoscale features in this area^11^.

In July 2017 a sub-mesoscale filament was detected on satellite radar images due to the occurrence of a nearly straight 50 km long and 500 m wide streak of sea ice in the MIZ of Fram Strait. The filament was formed by a strong salinity gradient between Atlantic and Polar waters close to the ice edge. High-resolution physical measurements revealed a frontal system of two strong currents flowing in opposite directions along the filament, leading to a horizontal inward-flow from both sides and mixing mostly in the upper 100 m and subduction inside the filament^11^.

The aim of this study was to highlight the biological response to physical forcing in this sub-mesoscale filament in Fram Strait. In order to accomplish this aim, we used a combination of automated underway-sampling and point sampling to collect samples for 18S meta-barcoding and optical data, addressing horizontal and vertical differences in the plankton composition in parallel to the physical measurements. The underway-sampling was accomplished with a resolution of ~ 2.5 km, which is adequate to resolve sub-mesoscale linkages between the physical environment and plankton composition^11^. This high-resolution information on spatial variability in plankton composition was complemented by a high-resolution information on total Chl *a*-biomass and the contribution of major phytoplankton groups obtained from hyperspectral underway spectrophotometry^17^. Additionally, data from sampling with a CTD-rosette and an optical zooplankton recorder, the LOKI (Lightframe On-sight Key species Investigation system)^18^ provided depth-resolved information on vertical plankton distribution in vicinity of the filament.

## Material and methods

### Description of the sub-mesoscale filament

In late July 2017 a ~ 7 km wide sub-mesoscale filament occurred under a 50 km long and only 500 m wide nearly straight streak of sea ice at 2.5°E/79°N in the marginal ice zone of Fram Strait. It was characterized by a thin surface meltwater layer above a layer of denser water, which extended to > 250 m depth^11^. Outside the filament, meltwater occupied the upper 15 m of the water column, leading to a strong stratification of the surface ocean. Here, Polar water was located directly below the meltwater layer at 20–40 m depth, while Atlantic water occupied the deeper water layers below 50 m. Inside the filament, the meltwater layer was thicker and occupied the top ~ 25 m, below which Atlantic water was found. High-resolution physical measurements revealed a frontal system of two strong currents flowing in opposite directions along the filament, leading to a horizontal inward-flow from both sides and mixing mostly in the upper 100 m inside the filament. Furthermore, the authors hypothesized that denser waters at ca. 100 m in the filament were in the process of subduction^11^.

### Sampling

**Figure 1:**
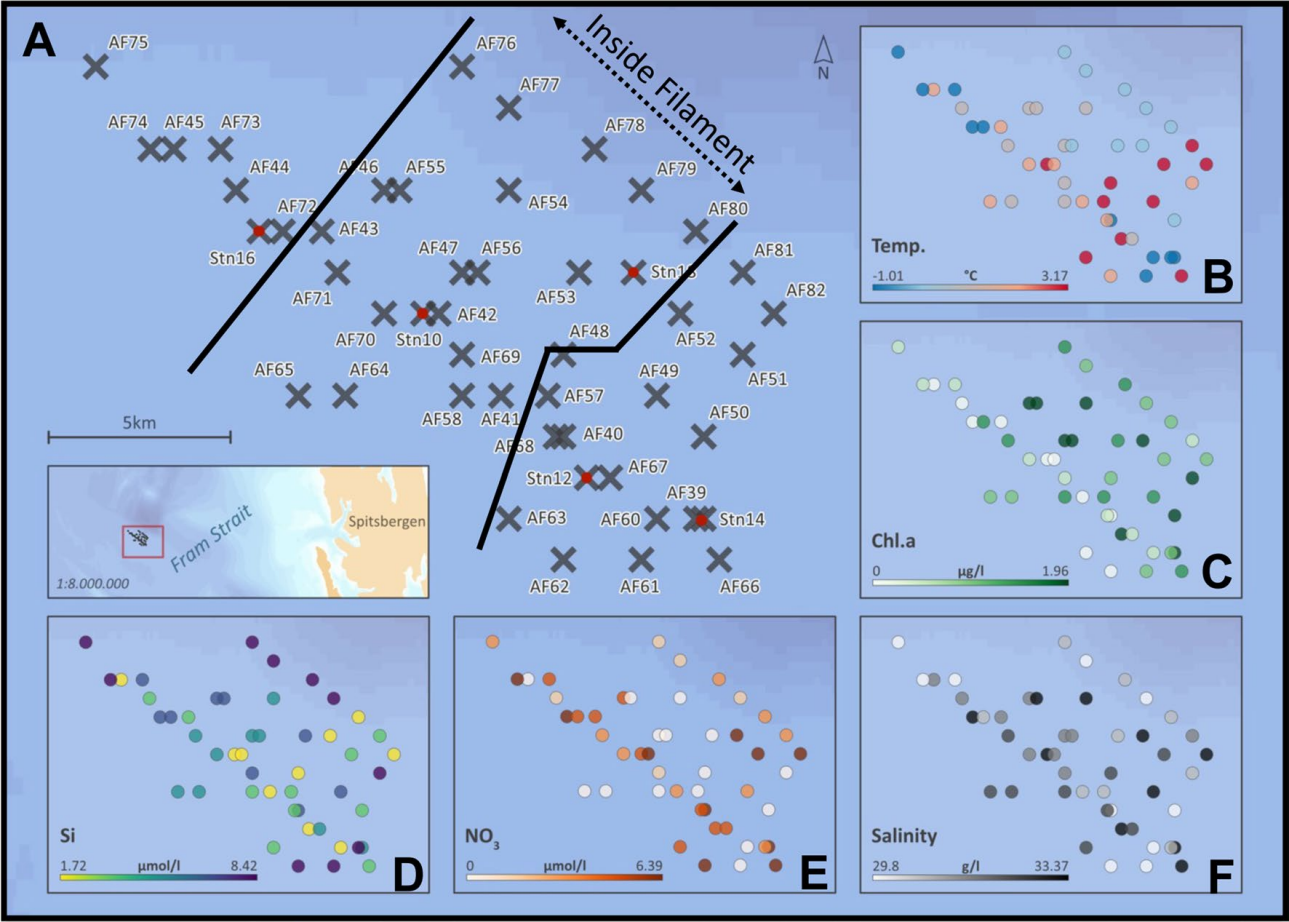
Map of study area in the Fram Strait (Arctic Ocean) reflecting the sampling-sites in the sub-mesoscale filament (Cross = AUTOFIM stations; Cross with red dot = CTD stations) (**A**), and concentrations of associated environmental parameters at the sampling sites (**B:** temperature; **C:** Chl *a*; **D:** silicate; **E:** nitrate; **F:** salinity).

The samples were collected in July 2017 during the RV Polarstern cruise PS107 in Fram Strait (Fig. 1). To address horizontal variability in plankton biodiversity samples were collected on one hand underway on five transects with a spatial resolution of ~ 2.5 km (1.5 nm) at a depth of ~ 10 m with the Automated filtration device for marine microorganisms AUTOFIM^15^, permanently installed on RV Polarstern. Two liters of seawater were collected and filtered on a 45 mm diameter Isopore Membrane Filters with a pore size of 0.4 µm (Millipore, USA) with max. 200 mbar. Additionally at five selected sites across the sub-mesoscale filament, samples from deeper water layers were taken with a rosette sampler equipped with 24 Niskin bottles (12 L per bottle) and sensors for Chl* a* fluorescence, temperature and salinity (CTD). Samples collected with the rosette were taken during the up-casts at 10, 20–30, 50, 100, 200 and 400 m depth. Subsamples of 2 L were transferred from the Niskin bottles into PVC bottles. Particulate organic matter for molecular analyses was collected by sequential filtration of each water sample through three mesh sizes (10 µm, 3 µm, 0.4 µm) on 45 mm diameter Isopore Membrane Filters at 200 mbar using a Millipore Sterifil filtration system (Millipore, USA). Subsequent to filtration, particulates are stored at − 80 °C until further processing in the laboratory.

### Environmental parameters

Data for temperature and salinity are available via PANGAEA (doi.org/10.1594/PANGAEA.894189; doi.org/10.1594/PANGAEA.889535). Samples with a volume of ~ 50 ml were taken in parallel to AUTOFIM sampling from the ship's pump system and from the CTD. The samples were directly frozen at − 80 °C for subsequent nutrient analyses in the laboratory. Nutrients were analyzed with an Alliance Evolution III continuous flow autoanalyzer (Alliance Instruments GmbH, Freilassing, Germany). The water samples were measured unfiltered. Measurements were made simultaneously on five channels: Phosphate^19^, silicate^20^, nitrite, nitrate^21^ and ammonium^22^. All measurements were calibrated with a five nutrient standard cocktail (All from Merck, traceable to SRM from NIST) diluted in artificial seawater (ASW), and ASW was used as wash-water between the samples. Data were all standardized by the same in-house reference material obtained from CTD water bottles. For each run we checked our standards with Reference Material for Nutrients in Seawater (CRM 7602-a + CRM 7603a) produced by National Metrology Institute of Japan (NMIJ). Our standards and methods have been proven by inter-calibration exercises like the International Council for the Exploration of the Sea (ICES) and Quasimeme.

### Total Chl a biomass and fractions of phytoplankton groups

Chl *a* concentrations and other phytoplankton pigments were determined using the high-pressure liquid chromatography (HPLC) on samples collected in parallel to the samples collected for eDNA analyses^17,23^. These data were also used to develop an algorithm for obtaining a continuous dataset along the ship transect (at ~ 250 m resolution) of the concentration of Chl *a* and major phytoplankton pigments from underway hyperspectral spectrophotometry^17,24^. First, the HPLC pigment data was used to calculate the fraction of each major phytoplankton on the total phytoplankton Chl* a* concentration (total Chl* a*) by applying the diagnostic pigment (DP) analysis developed previously by Vidussi^25^ and refined by Uitz^26^. With this method for each group its Chl *a* concentration was calculated from the weighted concentration of its specific DP which is for diatoms, prymnesiophytes, dinoflagellates, chlorophytes (which include the prasinophyte group), chrysophytes, cryptophytes, and prokaryotic microbes, respectively, fucoxanthin, 19-hexanoyl fucoxanthin, 19-butanoyl fucoxanthin, peridinin, zeaxanthin, and chlorophyll.The specific DP weights were determined by Uitz^26^ by applying multiple regression to the sum of all seven DPs versus the total chlorophyll-a concentration taking into account pigment measurements from a very large global data base. More details are provided in Uitz et al.^26^. Finally, the fraction of each phytoplankton group was calculated by taking it’s Chl *a* concentration multiplied by the total Chl *a* concentration. The total Chl *a* concentration was determined from the sum of monovinyl- and chlorophyllide *a* concentration.

In order to derive a high resolution surface Chl* a* concentration data of the four major phytoplankton groups (diatoms, dinoflagellates, prymnesiophytes and chlorophytes) identified in the HPLC data set within this study, we applied also the same diagnostic pigment analysis as for the HPLC data to the spectrophotometric pigment data set. For the three other phytoplankton groups no diagnostic pigments could be retrieved from the underway spectroscopy data, however, there contribution was found to be minimal for the Chl* a* in our study area (0–5%). We calculated the fraction of each phytoplankton group in respect to its Chl *a* compared to the total Chl *a* for both data sets.

### LOKI-casts and image analyses

LOKI casts were conducted from 400 m to the surface at each of the five stations, that were also sampled via CTD for eDNA analyses. The system continuously takes images (max. 19 frames sec^−1^) during the up-cast from mesozooplankton organisms that are concentrated by a 150 µm plankton net, leading to a flow-through chamber with a 6.1 mp camera (for a detailed description of LOKI see^18^). All images were loaded into the LOKI browser, a software that assigns optical parameters (hue factors, gray scale, skewness etc.) to each image and links the images to the respective metadata. Then, the image quality was enhanced and double takes of objects were removed using the software ZooMi. Subsequently, the images were uploaded to the EcoTaxa website (ecotaxa.obs-vlfr.fr), an application that facilitates the annotation, i.e. assignment of taxonomic categories, to the organisms presented on the images. Due to the high resolution of the LOKI images, it was often possible to identify families and sometimes-even species, thus tackling their fine scale distribution in the water column.

### DNA-isolation

Isolation of genomic DNA from the field samples was carried out using the NucleoSpin Plant Kit (Machery-Nagel, Germany) following the manufacturer’s protocol. The resulting DNA-extracts were stored at − 20 °C.

### Illumina-Sequencing 18S rDNA

For Illumina-Sequencing, a fragment of the 18S rDNA containing the hypervariable V4 region was amplified with the primer set 528iF(GCGGTAATTCCAGCTCC) and 964iR(ACTTTCGTTCTTGATYRR)^15^. All PCRs (polymerase chain reaction) had a final volume of 50 µL and contained 0.02 U Phusion Polymerase (Thermo Fisher, Germany), the tenfold polymerase buffer according to manufacturer’s specification, 0.8 mM (each) dNTP (Eppendorf, Germany), 0.2 µmol L^−1^ of each Primer and 1µL of template DNA. PCR amplification was performed in a thermal cycler (Eppendorf, Germany) with an initial denaturation (94 °C, 2 min) followed by 35 cycles of denaturation (94 °C, 20 s), annealing (58 °C, 30 s), and extension (68 °C, 30 s) with a single final extension (68 °C, 10 min). The PCR products were purified from an agarose gel 1% [w/v] with the NucleoSpin Gel Kit (Machery-Nagel, Germany) and Minelute PCR Purification kit (Qiagen, Germany). Subsequent to purification of the 18S rDNA fragment the DNA concentration of the samples was determined using the Quantus Fluorometer (Promega, USA). Prior to the library preparation, the DNA fragments were diluted with TE-buffer to a concentration of 0.2 ng/µL. The library preparation was based on the 16S metagenomic protocol of Illumina (Illumina, USA). Finally, sequencing of the DNA-fragments was carried out using a MiSeq-Sequencer (Illumina, USA). Raw sequences had an approximate length of ~ 200 bp. Sequences generated in this study have been deposited via GfBio^27^ in the European Nucleotide Archive (ENA) with the accession number PRJEB66268.

### Sequence analyses and annotation

Further bioinformatic processing of the raw sequences was done with the *dada2* package v.1.18 in R v.4.0^28^. Realignment of the sequences, as well as the removal of the forward and reverse primer, was done by the bioinformatic tool *Cutadapt* v.3.4^29^. To improve the evaluation of sequences, low-quality-3’ ends were trimmed based on a visual review of the quality plots^30^. By filtering the sequences based on the expected error, a minimum quality was guaranteed for sequence pairs. The remaining probable sequencing errors were identified using the error profile and rectified by *dada2*^28^. Remaining sequence pairs were merged to amplicon sequence variants (ASVs) by *dada2*, when having an overlap of at least 25 base pairs without a mismatch (Callahan et al. 2016). The *Protist Ribosomal Reference database* (PR2) was used to taxonomically classify each ASV^31^. Potential chimeres, caused by the polymerase chain reaction, were discarded^28^. Further statistical analyses were performed using R v.4.0.3 (R Core Team, 2021) in R Studio v.1.2.5001 (R Studio Team, 2019). The *rrarefy* function from the *vegan* package v.2.5–7 was used for rarefaction and normalization^32^. Visualizations and further calculations were done with *ampvis2* v.2.6.7*, ggplot2* v.3.3.5*, FactoMineR* v.2.4*, factoextra* v.1.0.7.999*, cluster* v.2.1.4 and *vegan*^33–37^. Analysis of similarities was done for the different cluster (I–IV) using the *anosim* function of the *vegan* package. Additionally, testing for significant differences of the environmental parameters inside the filament (IF), outside east of the filament (OE) and outside west of the filament (OW) was done using the *aov* function performing a one-way-anova. The scripts for the R analysis can be found on github (https://github.com/JoFrieWeiss/Weiss_Metfies_et_al_2023/tree/main). Further editing (e.g.increasing the size of text and legends in graphs) were performed with Inkscape (version 1.1.1). The mapping of the stations as well as the nutrient distribution in the surface samples was done using QGIS (QGIS.org, 2022). The significance level was set at p < 0.05 for all calculations.

## Results

### Environmental conditions

Based on the high-resolution physical characterization of the sub-mesoscale filament^11^, we grouped the samples collected in an area inside the filament (IF), outside East and outside West of the filament (OE and OW). The area inside the filament includes a corridor of ~ 7 km along the 500 m wide streak of sea-ice (Fig. 1).

**Figure 2:**
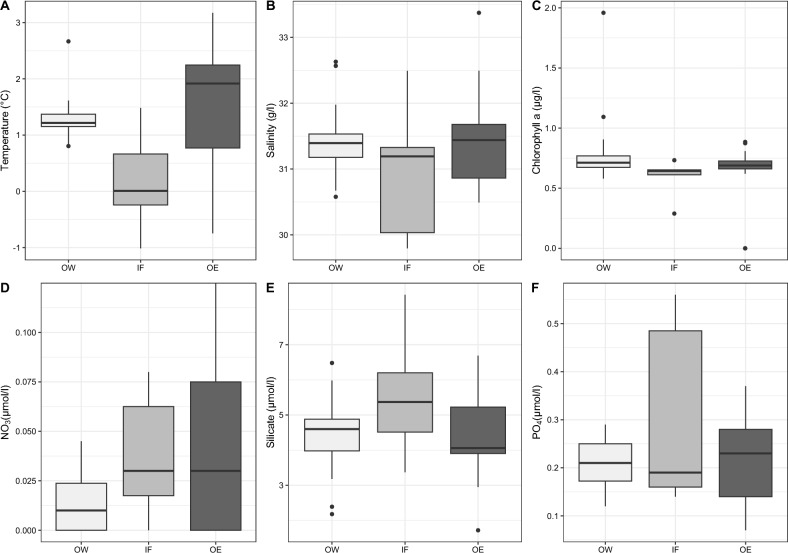
Chl *a *and nutrient concentrations measured at a depth of ~ 10 m in the study area (**A:** Temperature; **B:** Salinity; **C:** Chl a; **D:** Nitrate; **E:** Silicate; **F:** Phosphate). OW: outside west of the filament; IF: inside filament; OE: outside east of the filament.

Environmental parameters, including temperature, salinity, and concentrations of nutrient- and Chl* a* differed in surface waters at a depth of ~ 10 m between samples collected inside and outside the filament (Fig. 2). Temperatures outside west of the filament (OW) were significantly lower than inside the filament (p = 0.0068), while temperatures outside east of the filament (OE) were slightly higher than inside the filament. The temperature difference between outside west of the filament and outside east of the filament was also significant (p = 0.0008). Salinity values in the study area were not significantly different. West of the filament salinity values were slightly, but not significantly, lower than inside the filament (p = 0.1817), while the values east of the filament were comparable to the values measured inside the filament (p = 0.9988). In general, nitrate concentrations were low with maximum levels of 0.17 µmol/L in the entire study area, while silicate and phosphate concentrations were still quite high, pointing towards depletion of nitrate during a post bloom period. This situation is characteristic if non-silicifying taxa contribute significantly to the phytoplankton community subsequent to a diatom bloom (Fig. 4). The lowest nitrate levels occurred inside the filament. Photosynthetic biomass reflected by Chl *a* concentration was slightly lower in the surface layer inside the filament (Fig. 2). Looking at the phytoplankton group Chl* a* data, diatoms contribute between 39 and 70%, prymnesiophytes between 0 and 18%, dinoflagellates between 0 and 31%, chlorophytes between 5 and 46% to the total Chl *a*, while chrysophytes and prokaryotic phytoplankton have marginal contributions (0–4% and 1 to 2%). Supplement S1 provides an overview of the environmental parameter measured for this study and the assignment of samples to the different spatial areas identified across the filament.

### Vertical and horizontal plankton patterns in the sub-mesoscale frontal system

**Figure 3:**
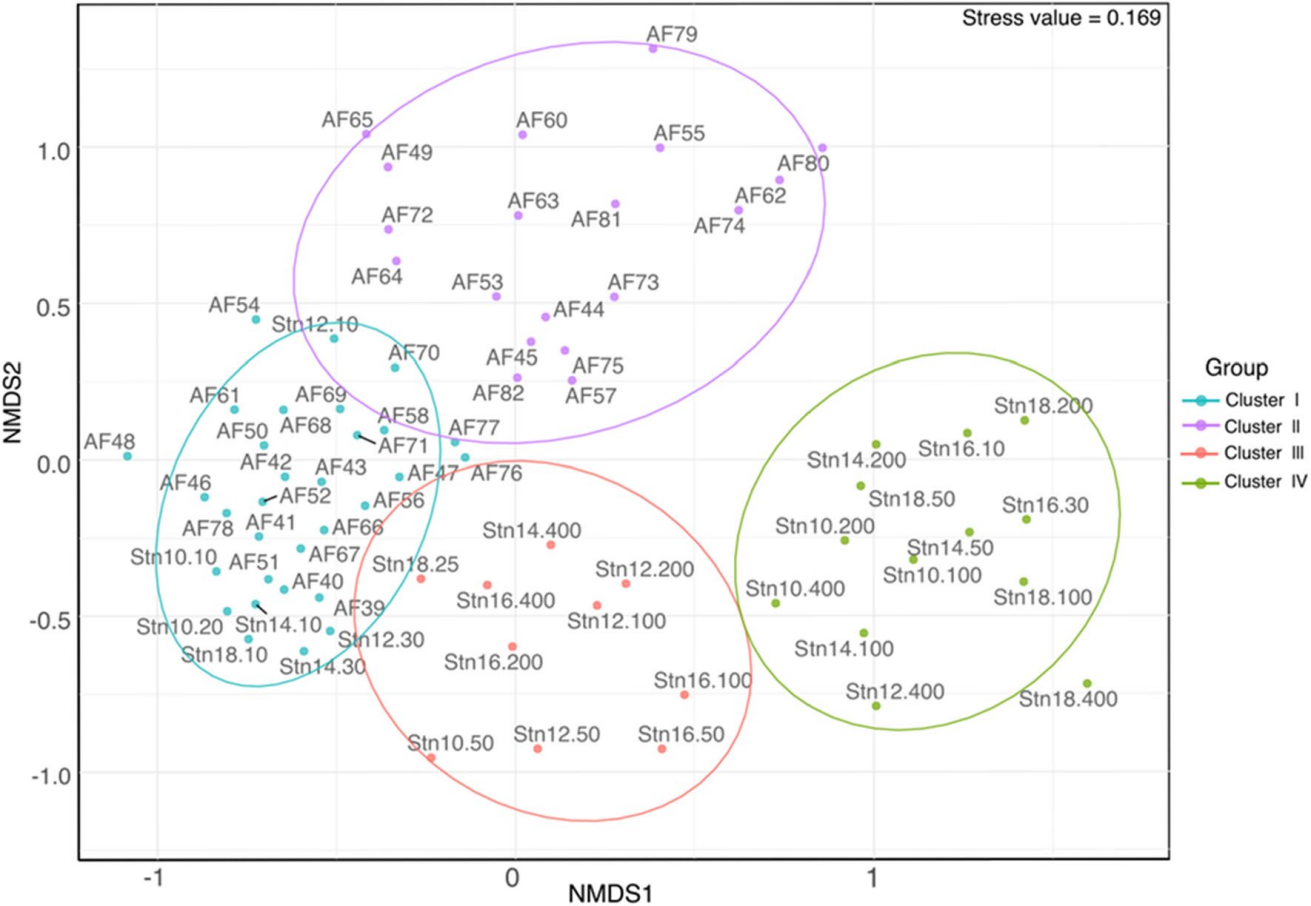
Non-metrical multidimensional scaling (nMDS-plot) of 18S community profiles in the sub-mesoscale filament displaying similarities between the eukaryotic plankton composition at different stations based on the Jaccard Index (Stress value = 0.169).

After quality filtering Illumina sequencing generated 5.058.797 high quality reads of the 18S rDNA V4-region from 74 samples collected near the surface on five different transects, and at six depths (10—400 m) at five CTD-stations across the sub-mesoscale filament. In a first step, community profiles were grouped based on Jaccard’s distances, into a non-metrical MDS ordination plot. There was a clear separation of 18S community profiles according to sampling depth and sampling location (Fig. 3). They grouped into four significantly distinct clusters (Fig. 3), supported by an ANOSIM (R = 0.81; p = 0.0001). Except for station 16, the 18S sequence composition in samples collected in the upper 30 m of the water column (cluster I and II) was significantly different from samples collected below (cluster III and IV), and significantly different between inside (cluster I) and outside (cluster II) of the filament. Cluster I contains samples collected mainly inside the filament, while cluster II contains samples outside the filament.

**Figure 4:**
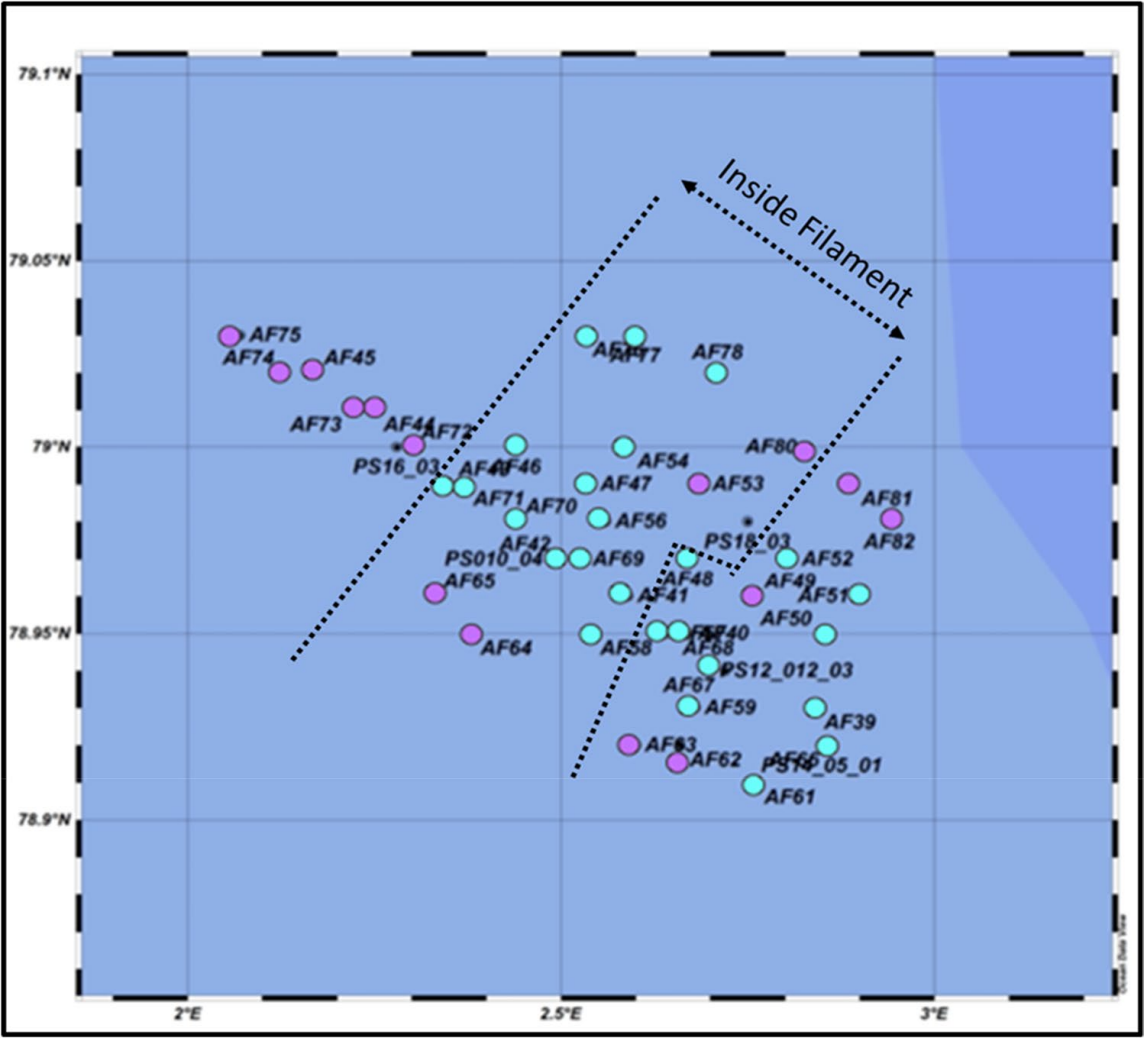
Assignment of cluster affiliations of samples to their positions in the study area. The different colors reflect the grouping in the nMDS-plot (turquoise: Cluster I; lilac: Cluster II).

This clustering was the basis for determining the horizontal expansion on the biological response on physical forcing across the sub-mesoscale filament. Similar to the physical characterization of the study area, we identified a corridor of ~ 7 km width along the sea ice streak in which the plankton community composition was different from that of the adjacent community around ~ 2 km away (Fig. 4). The samples in cluster III originated from water-depths between 50 and 100 m, and samples in cluster IV were collected at most stations between 100 and 400 m. The linkage between the clustering of samples and the physical environment in the observation area is further illustrated in a principal component analyses provided in the supplements (S3).

### Taxonomic composition of Eukaryotic plankton communities

**Figure 5:**
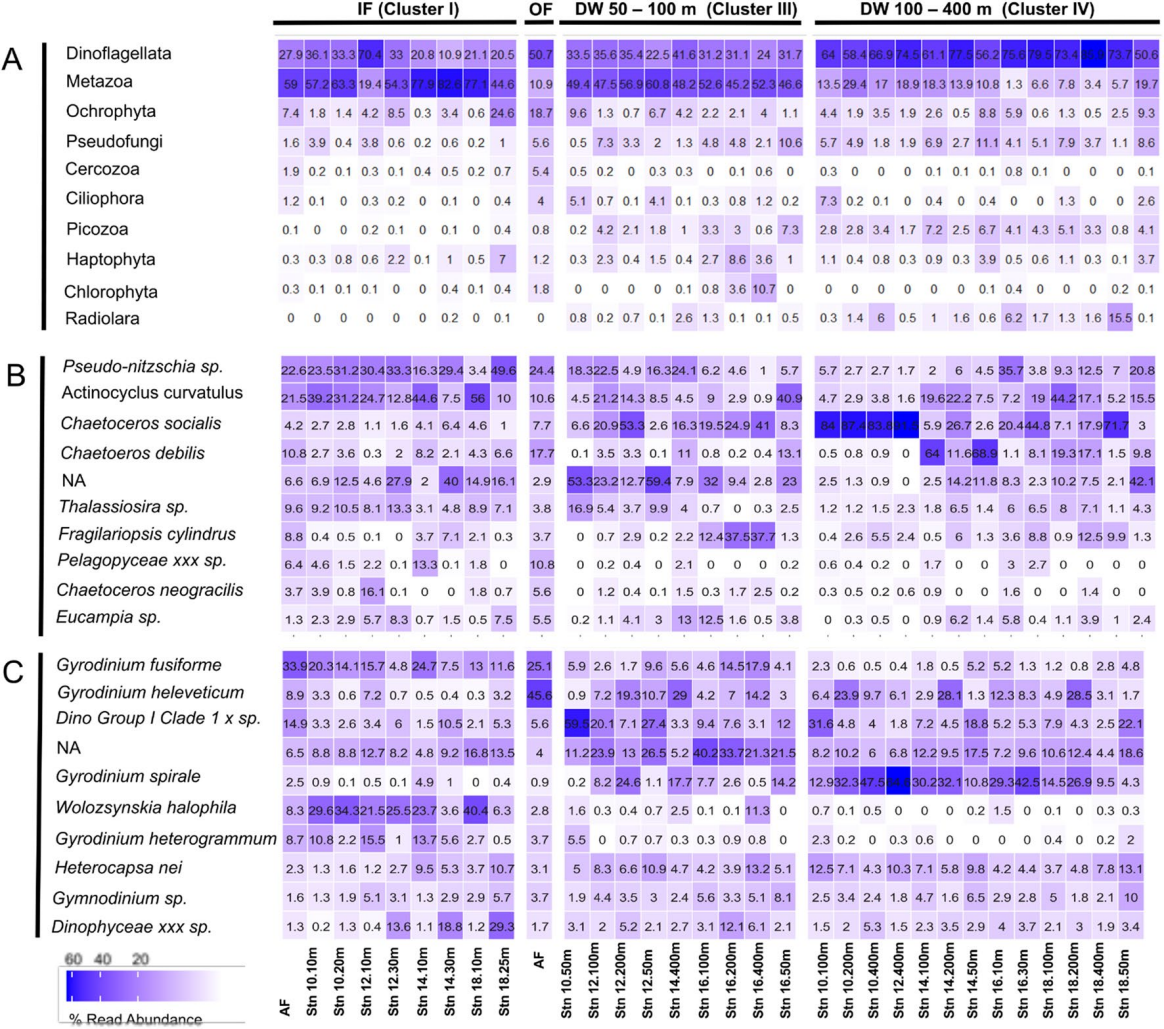
Relative sequence abundances (**A**) Higher taxonomic groups; (**B**) Ochrophyta; (**C**) Dinoflagellata.

Metazoa, Dinoflagellata and Ochrophyta constituted more than 50% of the eukaryotic microbial sequences in all samples of this study (Fig. 5A). Differences in the relative sequence abundance of these three taxonomic groups best explain segregation of the plankton community profiles in the MDSplot. The relative sequence abundance of Metazoa was highest in the upper 30 m of the water column inside the filament (p = 0.001), while sequence abundances of Dinoflagellata and Ochrophyta (mainly Bacillariophyta in this data set) were lower in the upper water column inside the filament compared to that outside the filament (p = 0.001). These data are supported by characteristic pigment data providing information on the contribution of different taxonomic groups to Chl *a* biomass. In cluster I diatoms and dinoflagellates were the main contributors to Chl *a* biomass, where diatoms contributed 40–60% and dinoflagellates 25–35% to total Chl *a* biomass. In cluster II diatoms contributed similarly as in cluster I to Chl* a*, but here, chlorophytes were more abundant, making up between 20 % (east of the front) to 50% (west of the front). The rest of the Chl* a* (~ 10% each) outside the filament was split up between dinoflagellates (mostly only east of the front) and prymnesiophytes (Supplement S2).

A detailed overview of sequence abundances of all samples collected via AUTOFIM is provided in the supplements (S4).

**Figure 6:**
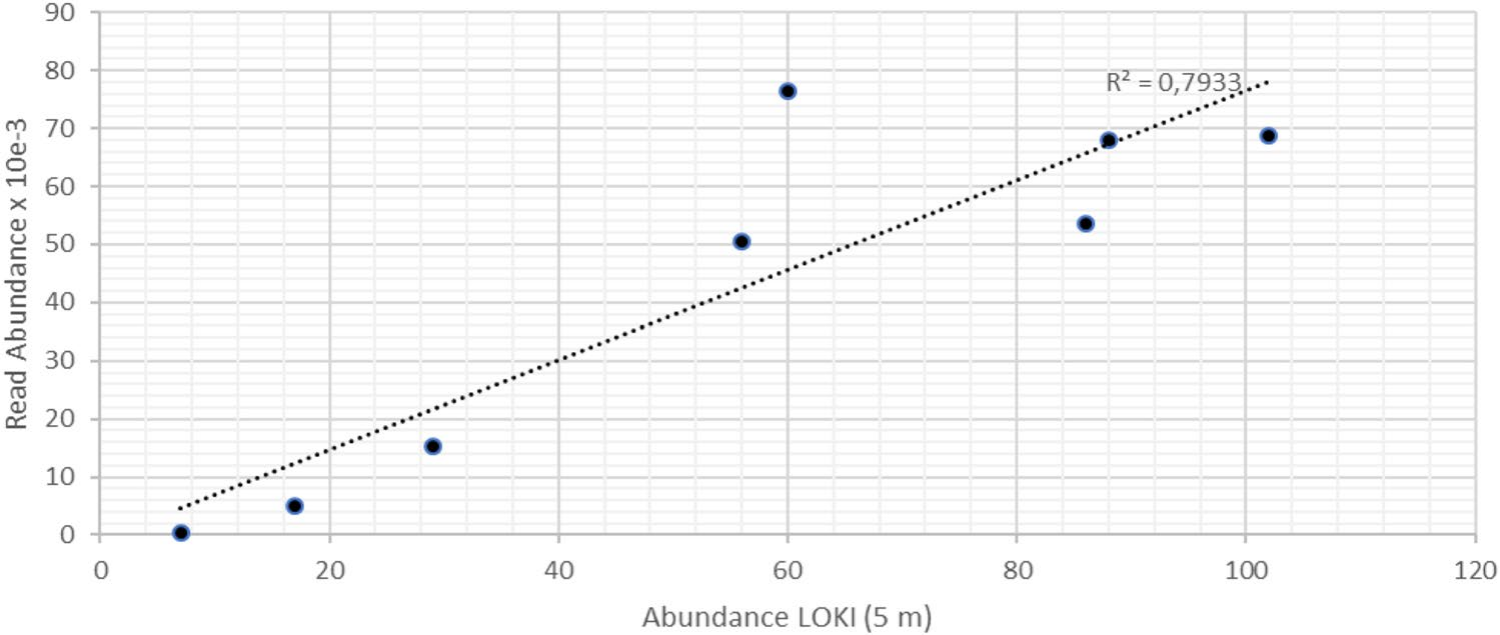
Correlation of optical counts of Zooplankton via the LOKI and eDNA analyses (Metazoan read abundance) for the upper 30 m at the CTD-stations.

Metazoan eDNA read abundances consisted mainly of the crustacean class Maxillopoda and the genera *Calanus*, *Oithona*, *Metridia* and *Pseudocalanus*. The use of LOKI allowed us for the first time to directly correlate relative sequence based information on zooplankton composition and distribution with optical counts. The metazoan read abundances were highly correlated to optical surveys of zooplankton abundances via the LOKI (Fig. 6). This is reflecting that relative changes ineDNA sequence abundances between the samples are tightly correlated with relative changes in species abundances. Moreover, the qualitative zooplankton composition was similar as derived from molecular and optical data. Both approaches thus confirm microscopic counts from multi net samples, which show higher zooplankton abundances in the upper 30 m inside the filament compared to outside the filament^4^.

Among the Bacillariophyta, the relative sequence abundance of the sea ice related pelagic diatoms *Actinocyclus curvatulus and Fragilariopsis cylindrus* was higher in the upper water column inside the filament than outside the filament (p = 0.001). Inside the filament, at stations Stn 10 & Stn 12, the relative sequence abundances of *A. curvatulus* in the upper 10 m and in the Chl *a* max were in a similar range, while it was lower in the Chl *a* max than in the upper 10 m at Stn 14 outside the filament. In contrast, *Chaetoceros socialis* appeared with low relative sequence abundances in the upper water column inside the filament as compared to both, outside the filament and deep water layers at stations Stn 10, Stn 12, Stn 14 and Stn 18 (p = 0.017). This species is known to be a major contributor to ice-free Arctic phytoplankton communities^38^. It contributed more than 80% of all Bacillariophyta at a depth of 400 m at Stn 10 & 12, inside the filament, corresponding to around ~ 8% of all sequences identified at this depth (Fig. 5B).

Among the Dinophyceae, the relative sequence abundance of *Gyrodinium fusiforme, Woloszynskia halophila* and *Pelagodinium beii* was the highest in the upper 30 m of the water column (p = 0.030) (Fig. 5C). The sequences of *Woloszynskia halophila* were significantly more abundant in the 10 m samples from inside the filament (p = 0.007), while *Gyrodinium heleveticum* sequences were significantly more abundant in the 10 m samples from outside the filament (p = 0.042) (Fig. 5C). *Gyrodinium fusiforme* sequence abundances were not significantly different between samples from inside and outside the filament.

Ciliophora, Cercozoa, Haptophyta (mainly *Phaeocystis sp.*), Picozoa, Choanoflagellida, Chlorophyta contributed less sequences to the 18 assemblages than Dinoflagellata and Ochrophyta, but their relative abundances also showed a distinct pattern, being lower in samples from the upper water column inside the filament, compared to samples from outside the filament. The relative sequence abundances of Haptophyta, Picozoa and Chlorophyta were alleviated in some of the samples collected below 100 m, suggesting enhanced physical export of these taxa that are known to have low sinking rates^39,40^. However, standard deviation in the deep community profiles was high (Fig. 5A).

## Discussion

There is growing evidence that marine physical features such as sub-mesoscale eddies or frontal systems impact ambient ecosystems, their biomes, and related biogeochemical processes by physical forcing that leads to changes in the availability of nutrients and/or distribution of organisms in a frontal system^3,4^. In this study, we combined for the first time high-resolution measurements of the physical environment with horizontally high-resolution surveys of molecular plankton diversity and Chl *a* biomass composition, and vertical profiles of physical and biological parameters for characterizing the effects of a marine physical feature on the surrounding ecosystem in the marginal ice zone of Fram Strait (Arctic Ocean). This unprecedented approach allowed us to estimate the spatial extent in which the planktonic community is influenced by a hydrographic sub-mesoscale structure. We observed significant horizontal and vertical differences in the 18S plankton community profiles and photosynthetic biomass that aligned with water mass distribution and physical forcing in the filament. Here, sea-ice melt related stratification might have been a key factor driving the community composition in the filament. Fram Strait is the main gateway for sea ice export from the Arctic Ocean, and large parts of the western Fram Strait are ice-covered throughout the year. The eastern Fram Strait is mostly ice-free and the southern location of the ice edge in this area is highly variable. In contrast to many other parts of the Arctic and Antarctic, the location of the ice edge is controlled dynamically in Fram Strait, i.e. it is primarily determined by the location and strength of the boundary currents (the West Spitsbergen Current transporting warm water northwards and the East Greenland Current transporting cold water and sea ice southwards). As a result, the variability of the sea ice edge between years and seasons is only approximately 50–100 km in Fram Strait while it may be up to 500 km latitudinal in other parts of the polar oceans, where the ice edge location is controlled thermodynamically through melting and freezing. It appears likely that submesoscale features such as filaments are also important in other marginal ice zones outside Fram Strait, but we note that the details of the physical and biological dynamics may be different between dynamically and thermodynamically controlled ice edges. In consequence, environmental conditions in the eastern Fram Strait are severely impacted by the presence or absence of the marginal ice zone (MIZ) and its related processes, such as sea ice melt and meltwater stratification^41^. The latter is supposed to be of central importance for setting patterns of Arctic phytoplankton distribution^42^. This is supported by the findings of this study, that describe enhanced sequence abundances of sea-ice associated diatoms, zooplankton and enhanced POC- export within the sub-mesoscale feature that was strongly stratified by meltwater occupying the upper 25 m inside the filament.

In general, the ASVs identified in this data set represented all major taxonomic eukaryotic microbial groups expected to occur in plankton samples in Fram Strait such as stramenopiles, dinoflagellates, Ciliophora, Haptophyta or Chlorophyta^43,44^. The physical–chemical settings provided an environment for the development of plankton communities, which were on one hand horizontally distinct inside and outside the filament. On the hand, they were vertically distinct, with surface communities being different from those in the underlying water layers. Phytoplankton communities have a regularly recurring annual succession pattern^45,46^. In Fram Strait, blooms of the diatom *Chaetoceros spp.* together with other centric diatoms are characteristic for pelagic phytoplankton blooms during early summer, followed by increasing abundances of small picoplankton such as Picozoa or Mammiellophyceae^46,47^. The higher presence of 18S sequences associated with *Chaetoceros sociales* outside the filament in conjunction with small picoplankton taxa and elevated biomass of Chlorophyta suggested the presence of a late bloom-community of pelagic origin at 10 m depth during the observation period in vicinity of the filament. Moreover, the presence of significant shares of *Chaetoceros socialis* sequences at a depth of 400 m only at stations inside the filament furthermore suggests enhanced export in this area of the filament via a combination of physical forcing amplifying the biological carbon pump. This assumption is in line with enhanced carbon flux inside the filament^3^, which was hypothesized to be a consequence of a subduction process ongoing in the area^11^. In contrast to the distribution pattern of *Chaetoceros socialis,* sequences of *Actinocyclus curvatulus,* had higher relative sequence abundance inside the filament compared to outside the filament. The occurrence of this species is tightly linked to the presence of sea ice^48^, suggesting it to be an indicator for a sea ice related community having elevated growth in the meltwater layer inside the filament, which went down to 25 m. Thus, Chl *a* biomass inside the filament might be associated with growth of sea-ice associated diatoms near the streak of sea-ice in the center of the filament.

Among the dinoflagellates, we found besides marine also fresh-water taxa, such as *Gyrodinium helveticum*^49,50^. One reason for the occurrence of freshwater species in the surface waters could be the melting of sea ice, which transfers fresh-water taxa from the melt ponds to the upper water column. This would also explain the higher relative abundance of *Woloszynskia halophila* inside the filament. This species is a brackish Dinophyceae species usually found in the Baltic Sea^51^, but also in sea-ice associated systems^52^. Although we find these fresh-water and brackish taxa, it is unclear if they stay alive under marine conditions or if we only detect the remains of the algae. The heterotrophic marine species *Gyrodinium spirale* found with higher sequence abundance inside the filament is often associated with diatom blooms in pelagic systems^53^. At high latitudes, sea-ice melt might serve as an iron source to promote phytoplankton blooms in its vicinity^54^, and it is known that heterotrophic *Gyrodinium* species, like *G. spirale* and *G. fusiforme*, prefer iron-enriched blooms^55^. Thus, we postulate that alleviated sequence abundance of *G. spirale* as well as higher sequence abundances of sea-ice related diatom species inside the filament might be related to higher sea-ice melt related iron concentrations in a small geographical area inside the filament. Moreover, iron-enriched blooms are preferential food sources for zooplankton^56^, which had alleviated sequence and species abundances inside the filament. In summary, the community composition inside the front system might indirectly suggest iron-intake from sea-ice near melting sea ice, though this study is missing information on iron concentrations in the study area. Future studies in the marginal ice-zone should assess the iron-flux from sea-ice to improve our mechanistic understanding of linkages between sea-ice melt and plankton productivity in the Arctic Ocean.

In this study the eDNA data for zooplankton correlated surprisingly well with the optical in situ surveys via LOKI. Here, it was certainly an advantage that LOKI captured the fine scale distribution of zooplankton which allowed to relate the zooplankton density within an interval from 5 m above to 5 m below each water sampling depth. Net samples integrate over much larger, pre-fixed intervals, and thus correlations between vertical point measurements in the water column and integrated net samples likely would have resulted in a much larger variation. The zooplankton taxa found in this study via eDNA analyses, such as *Calanus.*, *Oithona.*, *Maxillopoda*, *Metridia sp* and *Pseudocanalus.* are all well known as contributors to plankton communities in Fram Strait^4,57,58^, and corresponded well to the image data. Since we are aware that the 18S rRNA gene is of limited value for a detailed and reliable taxonomic identification of zooplankton, we did not carry out correlation analyses at species level. Our results nevertheless suggest that using the 18S rRNA gene has the potential to serve as a proxy for zooplankton abundances, and should thus be included in a smart holistic long-term observation strategy. Further evaluations of eDNA based zooplankton surveys with optical counts, however, are needed to test this conclusion.

In summary, our data suggest that sea ice melt and meltwater stratification is promoting elevated abundance and growth of ice-related taxa inside the filament. The presence and higher relative abundances of taxa, which are known to be tightly linked to high sea-ice concentrations, coincided with higher relative sequence abundance of metazoa. The distribution pattern of 18S eDNA sequences associated with metazoa are in line with the findings of the assessment of zooplankton composition via optical in-situ observations described in this study, but also net-tows that found higher abundances, of zooplankton inside the filament^4^. The concentration of zooplankton inside the filament in conjunction with the enhanced growth of sea-ice associated phytoplankton is likely the result of the convergence of surface water associated with the sub-mesoscale filament, which also led to the accumulation of sea ice^11^. Despite differences in sequence abundances of phyto- and zooplankton inside and outside the filament, Chl *a* concentrations at 10 m were only slightly different. Assuming zooplankton grazing, nearly similar Chl *a* concentrations inside and outside the filament might point towards higher primary productivity inside the filament, as suggested previously based on differences in nutrient concentrations^3^. The reproductive activity of the zooplankton inside the filament might be a consequence of the availability of sea-ice associated phytoplankton, which might have a better food-quality for zooplankton compared to phytoplankton dominated by *Chaetoceros *sp.^59^. Thus, alleviated carbon export inside the filament might be a combination of enhanced carbon export due to zooplankton grazing on sea-ice associated phytoplankton and enhanced export of biomass due to subduction processes inside the filament.

In conclusion, only by combining high-resolution eDNA analysis near the water surface, targeted vertical profiling at CTD stations and optical observations via the LOKI, we were able to assess the spatial extent of ecosystem change at both horizontal and vertical scales near the sub-mesoscale structure. We were able to show that changes in biology within the filament were closely linked to physical structuring and occupied a similar space. The high-resolution data from the upper water column showed a clearer picture of plankton community boundaries near the filament than the five CTD stations and the vertical optical surveys^3,4^. The latter, in turn, provided important information on the vertical distribution of plankton communities and carbon flux in the study area. By combining the information from the horizontal and vertical surveys, we assume that sea-ice melt and associated physical and biological processes in a sub-mesoscale filament may have led to elevated POC-export in an overall area of ~ 350 km^2^ of Fram Strait during summer of 2017, illustrating spatially far reaching consequences of sea-ice melt for marine ecosystems. This is consistent with previous observations from long-term sediment trap observations demonstrating higher export of POC in regions with seasonal sea ice cover or near the ice edge compared to ice-free regions. Here we have demonstrated the large spatial gradients that can persist also at the community level in the marginal ice zone. However, further high-resolution studies in the marginal ice zone are needed to make more precise and quantitatively upscaled estimates of spatial impacts of near-ice-edge processes on marine ecosystems.

### Supplementary Information

Supplementary Information 1.

Supplementary Information 2.

Supplementary Information 3.

Supplementary Information 4.

Supplementary Information 5.

## Data Availability

Raw sequencing data are deposited in ENA under accession number PRJEB66268. All other data are available via PANGAEA or provided as part of this manuscript.
