# Supplementary material for: Unprecedented insights into extents of biological responses to physical forcing in an Arctic sub-mesoscale filament by combining high-resolution measurement approaches
Source: Sci Rep. 2024 Apr 8;14:8192. doi: 10.1038/s41598-024-58511-y (PMC11001927; doi:10.1038/s41598-024-58511-y)
Supplement: Supplementary file 1 — Supplementary Information 1. [file 41598_2024_58511_MOESM1_ESM.pdf]

Supplementary data 1: Metadata, nutrient concentrations, temperature and salinity of samples analysed in this study

Station	Position	Latitude	Longitude	Depth [m]	T [°C]	Salinity [g/l]	Sigma-theta	Chl.a [µg/l]	NO3 [µmol/l]	NO2 [µmol/l]	Si [µmol/l]	PO4 [µmol/l]	NH4 [µmol/l]
Stn10.10	IF	78° 58,577' N	002° 29,370' E	10	2,6661	32,5667	25,971	0,603	0,03	0,1	2,39	0,13	0,26
Stn10.20	IF			20	4,9143	34,4555	27,253	2,219	0,07	0,04	2,33	0,17	0
Stn10.50	IF			50	3,2205	34,8933	27,779	0,234	3,43	0,15	3,57	0,44	1,98
Stn10.100	IF			100	3,2783	35,0598	27,907	0,033	12,11	0,14	4,41	0,79	0,11
Stn10.200	IF			200	2,4762	35,0169	27,946	0,009	12,21	0,05	4,85	0,79	0
Stn10.400	IF			400	0,9637	34,9361	27,997	0,014	12,76	0,04	5,68	0,89	0
Stn12.10	OE	78° 56,681' N	002° 42,085' E	10	3,1745	32,4934	25,869	0,885	0,06	0,04	1,72	0,09	0,17
Stn12.30	OE			30	5,1567	34,7644	27,470	0,899	3,95	0,11	3,56	0,34	1,26
Stn12.50	OE			50	4,0387	34,9269	27,724	0,188	7,2	0,27	3,77	0,54	1,48
Stn12.100	OE			100	3,776	35,0716	27,867	0,037	11,91	0,3	4,52	0,69	0,41
Stn12.200	OE			200	3,1432	35,0616	27,922	0,004	10,44	0,04	3,83	0,63	0,41
Stn12.400	OE			400	1,5734	34,9846	27,994	0,006	11,56	0,04	4,76	0,67	0
Stn14.10	OE	78° 55,581' N	002° 51,138' E	10	-0,747	33,3724	26,824	0,874	1,37	0,03	4,15	0,28	0,17
Stn14.30	OE			30	0,5509	34,3681	27,565	0,612	7,05	0,1	3,73	0,54	0,43
Stn14.400	OE			400	4,2524	34,9479	27,718	0,002	11,8	0,05	4,17	0,71	0
Stn14.50	OE			50	4,7323	35,0521	27,747	0,069	12,19	0,07	4,3	0,73	0,35
Stn14.100	OE			100	3,8796	35,058	27,845	0,016	12,07	0,05	4,37	0,72	0,16
Stn14.200	OE			200	3,4218	35,0669	27,899	0,006	12,74	0,04	5,92	0,76	0,1
Stn16.10	OW	79° 00,266' N	002° 16,940' E	10	-1,013	32,4913	26,120	0,289	6,39	0,07	5,37	0,56	0,2
Stn16.30	OW			30	-1,5518	33,9463	27,317	0,184	7,88	0,09	3,76	0,58	0,22
Stn16.50	OW			50	-1,2092	34,1918	27,506	0,083	9,09	0,1	4,71	0,63	0,39
Stn16.100	OW			100	3,8858	35,0242	27,818	0,101	12,09	0,11	4,27	0,74	0,12
Stn16.200	OW			200	3,5154	35,0608	27,885	0,005	12,08	0,05	4,24	0,73	0,18

Stn16.400	OW			400	2,1757	34,9836	27,946	0,001	12,48	0,07	5,16	0,76	0,18
Stn18.10	IF	78° 59,168' N	002° 45,451' E	10	1,1571	32,629	26,129	1,959	0,14	0,04	2,18	0,2	0,51
Stn18.25	IF			25	2,5615	34,4296	27,468	2,879	3,85	0,07	2,81	0,36	0,43
Stn18.50	IF			50	3,7612	34,9275	27,753	0,119	8,89	0,2	3,63	0,64	0,78
Stn18.100	IF			100	4,1043	35,0915	27,848	0,011	9,18	0,04	3,08	0,63	0,32
Stn18.200	IF			200	3,461	35,0696	27,897	0,006	10,43	0,02	3,7	0,69	0,15
Stn18.400	IF			400	1,1299	34,8994	27,957	0,002	12,66	0,05	5,93	0,86	0
AF39	OE	78°92,900' N	002°83,6206' E	11	0,0978	30,915	24,805	0,6893	0,025	0,01	6,43	0,34	0
AF40	OE	78°95,084' N	002°66,6276' E	11	0,5318	30,4929	24,446	0,6195	0,07	0,01	5,22	0,32	0,12
AF41	IF	78°96,456' N	002°58,90389' E	11	1,384	31,1866	24,957	0,588	0,02	0	3,2	0,17	0,05
AF42	IF	78°97,608' N	002°50,5329' E	11	1,6157	31,2334	24,980	0,5796	0,17	0,02	3,18	0,16	0,01
AF43	IF	78°99,692' N	002°35,824' E	11	1,464	31,2452	24,999	0,5802	0,035	0,01	4,04	0,19	0
AF44	OW	79°01,165' N	002°25,3752' E	11	1,253	31,2915	25,049	0,6135	0,01	0	3,94	0,16	0,02
AF45	OW	79°02,298' N	002°17,339' E	11	1,4831	31,3612	25,091	0,6096	0	0,01	3,37	0,14	0,08
AF46	IF	79°00,542' N	002°44,3289' E	11	1,3556	31,4321	25,156	0,7786	0,045	0,015	4,76	0,26	0,04
AF47	IF	78°99,193' N	002°54,4063' E	11	1,2579	31,387	25,126	0,798	0	0,02	4,44	0,25	0,51
AF48	OE	78°97,490' N	002°67,3831' E	11	2,283	31,5247	25,167	0,709	0	0	3,09	0,08	0,05
AF49	OE	78°96,331' N	002°79,1854' E	11	2,6908	31,7389	25,307	0,7243	0,015	0,015	5,23	0,21	0,23
AF50	OE	78°95,224' N	002°85,0963' E	11	0,858	30,7436	24,630	0,8089	0	0,02	4,01	0,11	0,18
AF51	OE	78°96,724' N	002°90,03' E	11	1,3668	31,1564	24,933	0,7971	0	0,02	6,61	0,26	0,07
AF52	OE	78°97,782' N	002°81,6124' E	11	2,0794	31,6348	25,270	0,71	0,08	0,02	3,94	0,07	0,06
AF53	IF	78°99,477' N	002°69,0313' E	11	1,1774	31,1737	24,959	0,713	0	0,01	5,15	0,12	0
AF54	IF	79°00,615' N	002°59,91' E	11	1,1974	31,9763	25,602	1,093	0	0,02	4,54	0,25	0,12
AF55	IF	79°01,049' N	002°45,5822' E	11	1,3594	31,6741	25,350	0,877	0	0	4,85	0,27	0
AF56	IF	78°98,734' N	002°56,476' E	11	1,1523	31,4016	25,143	0,9047	0	0,02	4,74	0,25	0,15
AF57	OE	78°95,844' N	002°64,8964' E	11	1,9525	31,2066	24,936	0,7271	0	0,01	4,04	0,19	0,02

AF58	IF	78°95,880' N	002°54,0541' E	11	1,2159	31,5341	25,246	0,7207	0	0,01	3,96	0,21	0,01
AF60	OE	78°92,969' N	002°78,9437' E	11	0,6848	30,7755	24,665	0,6714	0	0,03	2,95	0,14	0,29
AF61	OE	78°91,827' N	002°76,5331' E	11	0,0813	30,5385	24,502	0,6783	0	0,025	6,35	0,37	0,17
AF62	OE	78°92,057' N	002°66,7325' E	11	1,3955	30,8091	24,653	0	0,085	0,015	6,69	0,37	0,14
AF63	OE	78°92,836' N	002°60,4714' E	11	2,5131	31,5212	25,147	0	0,055	0,01	4,06	0,23	0,1
AF64	IF	78°95,648' N	002°38,9217' E	11	1,2188	31,4574	25,184	0,7119	0	0,01	4,09	0,21	0,04
AF65	IF	78°96,470' N	002°33,2619' E	11	1,3763	31,5753	25,269	0,6918	0	0,02	4,23	0,22	0,13
AF66	OE	78°92,464' N	002°86,7262' E	11	2,2102	31,8611	25,442	0,7262	0,14	0	4,06	0,28	0,21
AF67	OE	78°94,457' N	002°72,5941' E	11	1,3508	31,439	25,162	0,6686	0,05	0	4,55	0,24	0
AF68	OE	78°95,485' N	002°65,6674' E	11	1,9162	31,5395	25,205	0,6361	0,03	0	4,01	0,18	0
AF69	IF	78°97,111' N	002°53,8258' E	11	1,2014	31,3546	25,103	0,6728	0,01	0	4,89	0,21	0
AF70	IF	78°98,496' N	002°44,4012' E	11	1,5475	31,4113	25,127	0,6245	0,02	0	4,66	0,18	0
AF71	IF	78°99,272' N	002°37,9689' E	11	1,2781	31,5228	25,233	0,7317	0,02	0	4,73	0,17	0
AF72	OW	79°00,323' N	002°30,767' E	11	-0,2881	31,1926	25,044	0,7327	0,045	0,005	5,09	0,16	0,05
AF73	OW	79°01,554' N	002°22,6766' E	11	-0,1969	30,1907	24,232	0,6529	0,03	0,01	5,41	0,19	0
AF74	OW	79°02,341' N	002°13,5678' E	11	0,0752	29,7963	23,904	0,6511	0,08	0,01	8,42	0,5	0,58
AF75	OW	79°03,619' N	002°07,4702' E	11	0,0075	29,8758	23,971	0,6431	0,025	0	6,99	0,47	0,55
AF76	IF	79°03,951' N	002°53,8604' E	11	0,8046	31,0008	24,841	0,7336	0,005	0,005	6,48	0,29	0
AF77	IF	79°03,405' N	002°60,4599' E	11	0,9922	30,5783	24,491	0,6742	0,01	0	5,98	0,25	0
AF78	IF	79°02,063' N	002°71,3804' E	11	0,9859	30,9342	24,777	0,7401	0,025	0	5,59	0,23	0
AF79	IF	79°01,204' N	002°76,5639' E	11	0,8416	30,6728	24,575	0,6854	0,01	0,01	5,55	0,26	0,01
AF80	IF	79°00,149' N	002°83,9051' E	11	0,9613	30,9122	24,761	0,7062	0,02	0,03	3,5	0,13	0
AF81	OE	79°99,015' N	002°89,8559' E	11	2,1419	31,3471	25,036	0,6522	0,02	0	3,87	0,14	0,82
AF82	OE	78°98,254' N	002°94,0732' E	11	2,78	31,7199	25,285	0,6755	0,11	0	3,35	0,27	0
