# Supplementary material for: Unprecedented insights into extents of biological responses to physical forcing in an Arctic sub-mesoscale filament by combining high-resolution measurement approaches
Source: Sci Rep. 2024 Apr 8;14:8192. doi: 10.1038/s41598-024-58511-y (PMC11001927; doi:10.1038/s41598-024-58511-y)
Supplement: Supplementary file 2 — Supplementary Information 2. [file 41598_2024_58511_MOESM2_ESM.pdf]

Supplement S2:

Allocation of samples to the clusters in the nMDS plot and oceanographically distinct areas at the study site, timing and location of sampling, concentrations of total Chla and pigments specific for selected phytoplankton groups

Cluster nMDS	Sample	Grouping Filament	Date	Time (UTC)	Latitude	Longitude	Depth	Total Chl a [µg/l]	Chl a Haptophyta [µg/ml]	Chl a Diatoms [µg/l]	Chl a Chlorophyta [µg/l]	Chl a Dinoflagellata [mg/l]
Cluster_l	AF39	OE	29.07.2017	11:15	78,929002	2,836206	11	0,8801	0,33	0,59	0,08	0,00
Cluster_l	AF40	OE	29.07.2017	11:45	78,950835	2,666276	11	0,6665	0,41	0,52	0,07	0,00
Cluster_l	AF41	IF	29.07.2017	12:00	78,964562	2,590389	11	0,5074	0,46	0,45	0,08	0,00
Cluster_l	AF42	IF	29.07.2017	12:15	78,976081	2,505329	11	0,2716	0,36	0,48	0,16	0,00
Cluster_l	AF43	IF	29.07.2017	12:41	78,996906	2,35824	11	0,4285	0,47	0,42	0,11	0,00
Cluster_ll	AF44	OW	29.07.2017	13:01	79,013239	2,242007	11	0,5389	0,38	0,54	0,08	0,00
Cluster_ll	AF45	OW	29.07.2017	13:13	79,02298	2,17339	11	0,7454	0,38	0,54	0,08	0,00
Cluster_l	AF46	IF	29.07.2017	20:45	79,005419	2,443289	11	0,9716	0,16	0,56	0,08	0,20
Cluster_l	AF47	IF	29.07.2017	21:01	78,99193	2,544063	11	1,0349	0,08	0,57	0,08	0,27
Cluster_l	AF48	OE	29.07.2017	21:22	78,977004	2,656643	11	0,4523	0,27	0,41	0,12	0,21
Cluster_ll	AF49	OE	29.07.2017	21:40	78,963313	2,761854	11	0,4001	0,42	0,45	0,13	0,00
Cluster_l	AF50	OE	29.07.2017	21:55	78,952235	2,850963	11	0,4089	0,49	0,41	0,10	0,00
Cluster_l	AF51	OE	29.07.2017	22:08	78,967239	2,9003	11	0,7900	0,38	0,56	0,07	0,00
Cluster_l	AF52	OE	29.07.2017	22:21	78,976356	2,828067	11	0,6499	0,46	0,48	0,06	0,00
Cluster_ll	AF57	OE	30.07.2017	00:40	78,944477	2,698158	11	0,4510	0,24	0,49	0,13	0,14
Cluster_l	AF58	IF	30.07.2017	05:10	78,958799	2,540541	11	0,6993	0,01	0,61	0,12	0,27
Cluster_ll	AF59	OE	30.07.2017	05:11	78,958016	2,546614	11	0,9866	0,14	0,57	0,08	0,21
Cluster_ll	AF60	OE	30.07.2017	05:40	78,937229	2,707929	11	0,2614	0,32	0,51	0,17	0,00
Cluster_l	AF61	OE	30.07.2017	06:02	78,915426	2,755717	11	0,8365	0,02	0,74	0,09	0,15
Cluster_ll	AF62	OE	30.07.2017	06:10	78,915991	2,698643	11	0,6454	0,00	0,70	0,12	0,17
Cluster_ll	AF63	OE	30.07.2017	06:22	78,925939	2,623434	11	0,6778	0,00	0,65	0,11	0,24
Cluster_ll	AF64	IF	30.07.2017	07:00	78,956478	2,389217	11	0,6122	0,00	0,61	0,13	0,26
Cluster_ll	AF65	IF	30.07.2017	07:10	78,964702	2,332619	11	0,7850	0,00	0,59	0,11	0,30
Cluster_l	AF66	OE	30.07.2017	16:59	78,924641	2,867267	11	0,9351	0,14	0,33	0,09	0,44
Cluster_l	AF67	OE	30.07.2017	17:22	78,943013	2,737124	11	0,3647	0,37	0,17	0,14	0,31
Cluster_l	AF68	OE	30.07.2017	17:40	78,957057	2,640014	11	0,6306	0,19	0,45	0,11	0,25
Cluster_l	AF69	IF	30.07.2017	17:58	78,971105	2,538258	11	0,6232	0,11	0,35	0,11	0,42
Cluster_l	AF70	IF	30.07.2017	18:16	78,984959	2,444012	11	0,3362	0,35	0,13	0,15	0,37
Cluster_l	AF71	IF	30.07.2017	18:22	78,989445	2,409815	11	0,3880	0,28	0,17	0,14	0,42
Cluster_ll	AF72	OW	30.07.2017	18:42	79,003229	2,30767	11	0,4553	0,00	0,52	0,15	0,33
Cluster_ll	AF73	OW	30.07.2017	18:58	79,015537	2,226766	11	0,5401	0,05	0,58	0,13	0,25
Cluster_ll	AF74	OW	30.07.2017	19:14	79,026729	2,135678	11	0,4264	0,08	0,80	0,12	0,00
Cluster_ll	AF75	OW	30.07.2017	19:22	79,033776	2,092511	11	0,4020	0,13	0,78	0,09	0,00
Cluster_l	St10	IF	29.07.2017	17:40	78,977201	2,493866	10	0,5619	0,21	0,46	0,12	0,21
Cluster_l	St12	OE	30.07.2017	01:42	78,944696	2,70004	10	0,6769	0,27	0,51	0,10	0,12
Cluster_l	St14	OE	30.07.2017	11:16	78,926996	2,850603	10	0,6996	0,26	0,58	0,10	0,06
Cluster_lV	St16	OW	30.07.2017	22:13	79,00628	2,283849	10	0,5526	0,30	0,28	0,13	0,29
Cluster_l	St18	IF	31.07.2017	05:11	78,987103	2,750504	10	1,4596	0,10	0,64	0,08	0,18
