# Supplementary material for: Unprecedented insights into extents of biological responses to physical forcing in an Arctic sub-mesoscale filament by combining high-resolution measurement approaches
Source: Sci Rep. 2024 Apr 8;14:8192. doi: 10.1038/s41598-024-58511-y (PMC11001927; doi:10.1038/s41598-024-58511-y)
Supplement: Supplementary file 5 — Supplementary Information 5. [file 41598_2024_58511_MOESM5_ESM.pdf]

Supplement S5:

Number of zooplankton organisms on LOKI images and derived zooplankton abundance

Station	Latitude	Longitude	Depth [m]	Number of Zooplankton organisms [n] on LOKI images in 5 m intervals (Depth ± 2.5 m)	Zooplankton Abundance [n ind. m^{-3}]
Stn10.10	78° 58,577' N	002° 29,370' E	10	60	43
Stn10.20			20	102	73
Stn12.10	78° 56,681' N	002° 42,085' E	10	29	21
Stn12.30			30	86	61
Stn14.10	78° 55,581' N	002° 51,138' E	10	88	63
Stn14.30			30	56	40
Stn16.10	79° 00,266' N	002° 16,940' E	10	7	5
Stn16.30			30	17	12
